# Beyond Satisfaction: Person‐Centred Care and the Physical Environment Revisited—An Integrative Review

**DOI:** 10.1002/nop2.70395

**Published:** 2026-01-21

**Authors:** Amy‐Louise Byrne, Ainslie Hall, Ellie Cutmore, Jennifer Mulvogue

**Affiliations:** ^1^ School of Nursing, Midwifery and Social Sciences Central Queensland University Brisbrane Queensland Australia

**Keywords:** experience, healthcare design, patient satisfaction, person‐centred care, shared decision‐making

## Abstract

**Background and Aims:**

To explore literature in relation to the physical healthcare environment and person‐centred care to understand how it can be better supported at the system level.

**Methods:**

An integrative review using the work of Whittmore and Knafl. The review was designed around problem identification, literature search, data evaluation, data analysis and presentation of stages.

**Data Sources:**

CINAHL, Embase, PubMed and Scopus databases were searched, and 29 articles were included.

**Findings:**

Articles were considered in relation to their context, and themes for each were generated. These were: Aged care (The ‘Safety Gaze’, Reconciling safety to support an environment of personhood and Nature and stimulation); Paediatrics and Neonatal (Supporting parenthood, Person‐centred care (un)supported through control and Satisfaction and expertise); Maternity (Sterility versus person‐centred care and Controlling the birth experience); Acute care (Participation in the environment, First and foremost a healing environment, Decentralised nurses station and single rooms); Mental Health (Safety versus person‐centred care and Calming the environment); and Outpatient (Genuine access to information). The review found perceptions of safety and risk, and the idea of ‘hotel culture’, a negative connotation for healthcare staff. While person‐centred design in hospitals makes for a more aesthetically pleasing environment and positive experience, it is argued these elements are superficial in nature, rather than authentic.

**Conclusion:**

While described as person‐centred, many design elements were more about satisfaction than engagement and shared decision‐making. Healthcare policymakers, accreditors and leaders must move beyond satisfaction and consider environmental changes that genuinely engage people in their care and promote shared decision‐making.

**Implications for the Profession and/or Patient Care:**

What is already known? Person‐centred care is a requirement of health services. Person‐centred environmental changes have recently occurred to the healthcare environment. What this article adds? Many healthcare designs are more about satisfaction rather than genuine partnership. While aesthetics are important for experience, we must move beyond satisfaction to ensure that systems and environments supports person‐centred care.

**Reporting Method:**

PRISMA.

**Patient or Public Contribution:**

No Patient or Public Contribution.

## 1 Introduction

Quality health service delivery relies on person‐centred care (Australian Commission on Safety and Quality in Healthcare [ACSQHC] 2024a, 2024b). While definitions of person‐centred care exist and often share congruence, there is no agreed definition of the term (Byrne et al. 2021). Some early definitions emphasised practice (Lusk and Fater 2013; Castro et al. 2016), while more contemporaneous literature articulates the importance of governance, leadership and the health system (McCormack and McCance 2025). The Australian Commission on Safety and Quality in Healthcare (ACSQHC) defines person‐centred care as ‘care that respects and responds to the preferences, needs and values of patients and consumers’ (ACSQHC 2024a, 2024b, para. 1). In the absence of a clear definition, person‐centred care is often used to describe a ‘standard of care that ensures the patient is at the centre of care delivery’ (McCormack and McCance 2025), with central tenets including communication, shared decision making, and goal setting (ACSQHC 2025).

Criticism of person‐centred care suggests that new public management of healthcare frames the concept as a compliance measure, rather than genuine partnering and decision‐making (Byrne 2022). Additional criticisms centre on the Western foundations of person‐centeredness, which may not truly capture the cultural needs of Aboriginal and Torres Strait Islander people (McMillian et al. 2010). Nonetheless, the person‐centred care philosophy has been facilitated via several means, including shared decision‐making legislation in the United Kingdom (Coulter 2017), patient rights (e.g., ‘right to health’‐ Germany), and, at the local level in Australia, via hospital accreditation standards (ACSCHC 2021). This means person‐centred care is a requirement of modern healthcare.

More recent iterations of person‐centred care have been careful to articulate the importance of the care environment as a key element of person‐centred care, often outlining some of the organisational (or system) responsibilities. The care environment included the physical spaces and places, the safety and functionality, emotional atmosphere, and accessibility of a facility (Rodríguez‐Labajos et al. 2024; Kylen et al. 2024). For clarity, this article is concerned with the physical or built environment in which care is provided and how this supports person‐centred care. Features of person‐centred care environments include lighting, outdoor space, convenient access, wayfinding signage, accommodations for family and decentralised nursing (ACSQHC, n.d.). Other literature expresses that the healthcare environment can evoke emotions (Bogaert 2021) and has the ability to impact a person's level of participation in their health journey (Kvæl and Bergland 2021).

The physical environment can be well designed to improve patient health and reduce stress and injury to those receiving and providing care. Such elements include good ventilation, accessible handwashing facilities, appropriate transfer devices, centralised linen, reduced noise, use of natural light, safety and space (World Health Organization 2023). Other design features, such as purposeful colouring, aid in cognition and help distinguish visual information in the older person (Hencová and Kotradyová 2023). Indeed, for older people, the built environment can offer potential and limitations to their independence and to their individual expression (Fashold et al. 2024).

Direct care is commonly the focus of clinicians; however, there are often shortcomings when it comes to facility design, such as physical space and patient‐facing systems and processes impacting care provision (Berry et al. 2020). Thus, there is a close association between person‐centred practice and the role of the physical environment. Kang et al. (2022) found people admitted to intensive care units who perceived they had received person‐centred care were also more aware of their environment and surroundings, reported greater satisfaction with their experience, and were less frightened of treatment. Design features that reduce physical risks, allow people to be seen and see people, provide stimulation and support engagement are now premised in environmental audit assessments, such as the German Environmental Audit tool (G‐EAT), highlighting the importance of design and function in care environments (Fashold et al. 2022).

Undoubtedly, the physical environment in the healthcare system is an important element of care. However, how it fits within the person‐centred care concept requires further exploration.

## 2 Background

Recently, Byrne (2024) advocated for a separation of terms: person‐centred *care* as a system responsibility, and person‐centred *practice* as the way in which clinicians provide care. These terms are equally important and together form the basis for person‐centeredness. This separation is needed, as person‐centred care discursively positions the responsibility with health professionals and often neglects the systemic and structural challenges that create barriers to care and practice (Byrne et al. 2021). Under this lens, the time is right to revisit the physical environment and person‐centred care.

## 3 Methods

An integrative review, using the framework of Whittmore and Knafl (2005), was selected as it facilitates the summary of empirical and theoretical data (literature), while also building on theory, practice and policy. The review was designed around problem identification, literature search, data evaluation, data analysis, and presentation stages (Whittmore and Knafl 2005).

### 3.1 Problem Identification

This review aimed to explore literature in relation to the physical healthcare environment and person‐centred care to understand how it can be better supported at the system level. The question asked of the review was:

How is the current physical healthcare environment positioned to support person‐centred care, and what enhancements can be made at the system level?

While this enquiry will naturally consist of elements related to clinical practice, it focused on elements not specific to practice with the view of understanding how person‐centred care (as a system responsibility) can be better supported.

### 3.2 Search Strategy

The search strategy was developed using the modified PICo (Problem/population, Intervention and Context) framework (Hosseini et al. 2024). The use of PICo enabled the development of search terms across key domains, allowing for a cohesive and systematic search of literature. Table 1 demonstrates the domains and search terms used. A health service Librarian assisted with the search terms and strategy. Given the aim of the review was to assess person‐centred care across different healthcare contexts, we included adjacent terms such as women‐centred care and family‐centred care. While these are similar conceptually, they are nuanced in meaning and operationalization; however, when coupled with the built environment, the terms were suitably synonymous. An example search string is provided as Appendix A. Search strings were limited to title, abstract, and keyword (where possible).

**TABLE 1:** PICo search terms.

P	Problem/Population	Person‐centred care
I	Intervention/Interest	The physical environment
Co	Context	Healthcare settings
**Domains**		**Keywords and MeSH terms**
P	Person‐centred care	‘Person cent* care’ OR ‘patient cent* care’ OR ‘family cent* care’ OR ‘Client Cent* care’ OR ‘resident cent* care’ OR ‘woman cent* care’
I	The physical environment	‘Physical environment*’ OR ‘health* structure’ OR infrastructure OR surroundings OR ‘practice environment’ OR ‘facility design and construction’ OR ‘hospital design’
Co	Healthcare setting	Hospital OR ‘health clinic’ OR ‘health sector’ OR ‘nursing home’ OR ‘health service’ OR ward OR ‘inpatient service’

### 3.3 Data Evaluation

An inclusive approach to the practice environment and professional context was taken. The review excluded home and community‐based care, as the aim of the review was to explore the care environment within which people receive care (this includes inpatient, outpatient, hospital, and aged care environments). Person‐centred care includes the physical environment, as well as organisational systems, practices, and support structures. To control for variability of these concepts in the search terms used, the below inclusion and exclusion criteria were adopted (Table 2).

**TABLE 2:** Inclusion and exclusion criteria.

Inclusion	Exclusion
Peer reviewed, Full text, English language	
Published between 2000 and 2024, to capture the time period for which person‐centeredness was adopted into health services	Systematic reviews, scoping reviews, meta‐analyses, integrative reviews
Healthcare environments including inpatient (across any specialty)	Community health, for example home care
Includes analysis of the physical care environments and its impacts on person‐centred care. (Person‐centred care is defined as care which supports shared decision‐making, autonomy, dignity, privacy, respect, control, individualization, family, goal setting, outcomes important to the person)	Articles solely related to practice rather than the healthcare environment
	Articles related to technology, training, and education, as the focus was on the physical environment, rather than digital integration of systems

The Cumulative Index for Nursing and Allied Health Literature (CINAHL), Embase, PubMed, and Scopus databases were searched in October 2025. In the first‐round, a title and abstract review was completed by all members of the research team, with each article being independently reviewed by two team members. Conflicts for inclusion were resolved via a consensus meeting. Full text review was completed by all members of the research team, with each article being reviewed by two members independently. Conflicts were resolved via a consensus meeting of all team members.

Twenty‐six articles were selected for quality review using the Mixed Methods Appraisal Tool (MMAT) (Hong et al. 2018). Given that studies were not included or excluded based on methodology or methods, the MMAT was useful in determining the quality of each included study, allowing a rigorous assessment of the research question, study, and findings. No articles were excluded based on quality appraisal; thus, 29 articles were included in the review.

### 3.4 Data Analysis

As per Whittmore and Knafl (2005), analysis was completed by ordering, coding, categorising, and summarising the data. Data was ordered by care location (see Table 3), allowing analysis to be guided by the unique contexts of care (e.g., aged care). Initial analysis was completed by ALB and EC via inductive analysis, letting the data identify the initial codes. Second round categorisation within each context was generated via an iterative team approach involving all authors, where codes were grouped and categorised. Initial categories were collapsed into broader themes by consensus, again via the entire team. Finally, the themes of each context were considered as a whole, integrating the research problem back within the overarching context, as per the integrative review process (Whittmore and Knafl 2005).

**TABLE 3:** Summary of included articles.

Article	Citation	Contexts of care	Person‐centred care (or synonym) descriptor	Physical environment features which support PCC	Subtext
**Aged and Palliative care**					
1	Beuls et al. (2024)	Palliative care	Human centred Attune to their needs	Atmosphere of proximity Colours, spatial design Access	Safety User experiences
2	Boumans et al. (2022)	Residential aged care, The Netherlands	Autonomy, decision‐making	Enables freedom of movement (open doors). Private room and own key to access	Safety The physical environment facilitated access to family
3	Douglas and Douglas (2004)	Surgical, medical, elderly and maternity care in major NHS hospital, United Kingdom	Person/patient and family needs	Personal space, privacy, a homely feeling, meeting needs of visitors, noise, control, access to external areas, supporting communication, lighting, well planned ward, visually for safety, access to kettle, toaster, microwave. Light toilet seats, or curtains people with limited reach can shut	Safety, reduce stress and promote healing. Staff felt improved design came at a cost to clinical care
4	Chaudhury et al. (2017)	Residential aged care, Canada	Best practice in care, encouraging socialisation	Support functional ability, awareness and orientation, Safety and security, homeliness, sensory stimulation, social interaction, privacy and comfort	Improving autonomy, safety, socialisation
5	De Boer et al. (2018)	Residential Aged care, The Netherlands	Wellbeing, autonomy, privacy, comfort	Privacy, sensory stimulation (temperature regulation, daylight glare), view and nature, accessibility to outdoor areas, orientation, and routing. Communal cooking areas for sensory benefit	Safety of residents. Space is important but does not always facilitate use. Maximising orientation and cognition
6	Mobley et al. (2017)	Specialised care unit in residential aged care, USA	Personhood, holistic care, recognition, respect and trust	Common spaces, personalised spaces, stimulating interior design, spatial orientation via a H design, wayfinding, lighting	Aggression is mitigated, safety, reduced risk of absconding
7	Shannon et al. (2018)	Rural and remote hospitals in Australian	Respectful relationships between staff, family and the people they care for A calm presence A workplace in which staff are empowered to develop their practice	Allows for movement and continuous observation Close proximity to nurse's station. Calm and quiet Physical environment seen as not ‘safe’ for unsupervised wandering (exit doors opening to busy roads, high floors, stairwells posing a falls risk)	Nurses value patient safety and limiting distress Risk and restraint Nurses value collaboration with family to assist with reducing patients' anxiety and agitation
8	Van den Berg et al. (2020)	Geriatric ward in hospital, The Netherlands	Wellbeing, healing, enhanced cognitive function, independent functioning	Green (plant) spaces created throughout ward. Promoted a more relaxing, inviting environment. Improved functioning	Did not improve staff satisfaction and was at times seen as messy and an additional duty. ‘Less safe’ but had positive effects on patient wellbeing
**Paediatrics and Neonatal**					
9	Feeley et al. (2020)	Neonatal intensive care, Canada	Family‐centred care. Keeping family unit together, improving family autonomy and skills	Single family rooms allow for privacy and for families to remain together. Facilitated more engagement with care and perceptions of control. More discharge ready as they had time as a family to ‘practice’ their new baby skills	Family rooms facilitated greater respect for staff, mothers were less stressed
10	Kotzer et al. (2011)	Children's Hospital, USA	Person/Patient outcomes, satisfaction	Charting area, room layout, natural light, storage, comfort and appeal, privacy, security, wayfinding, parking, break room, waiting room, quiet space, cleanliness, furniture, noise, room as a healing environment	Safety and satisfaction with design
11	Pattabi et al. (2024)	Paediatric inpatient, Qatar	Fulfilling unique needs, preferences, and values	Colourful and attractive, lights, fun designs, spacious with room to move, toys and designated areas to play. Pictures on walls, cartoons Signage, consultation rooms, bedroom, television	Diverting attention away from clinical care, pain, and illness. Reduce anxiety and fear
**Maternity**					
12	Nielsen and Overgaard (2020)	Obstetric unit in regional hospital, Denmark	Person/Patient satisfaction, quality care, experience, respect, values and preferences, physical comfort, coordination and integration of care, information, communication, transition and continuity, family engagement and emotional support	Welcoming space, audio and visual stimulation, Nordic furniture, bathtub, relaxation area with sofa, room allows for partners to be involved in labour and delivery, positive distraction. Single room, lighting	Satisfaction, safety. Reduced anxiety
13	Sheehy et al. (2011)	Birthing unit in Hospital, Australia	Privacy, feeling safe and nurtured	Space, privacy, noise, bath, en‐suite, light, colour, texture, indoor environment, femininity, accommodation for support people. Midwives adjust room and equipment to support safety	Sterility versus homely, safety and comfort
**Acute Care**					
14	Annemans et al. (2016)	Day Surgery centers, Belgium	Experience, control	Having own room for day surgery, being wheeled into theatre or walking in. Being wheeled renders the person passive, but also protects them from the environment	Safety, power, passive or active role in care processes. Perception changes according to walked or wheeled
15	Astin et al. (2020)	Medical assessment unit, United Kingdom	Person/patient wellbeing, positive experience	Noises from equipment, banging doors, general ward noise, temperature	The biomedical importance of sleep on healing. The person/patient experience of the ward
16	Buchwald et al. (2024)	Palliative care in acute hospitals	Alleviate suffering and enhance quality of life Experience	Discreetly hidden medical apparatus Soothing natural elements Dimmable lighting Comfortable furniture Lively physical surrounds	Calming and soothing environment for the patient and the family Included excerpts around communication and interaction, but not specially related the physical environment
17	Catt and Giridharan (2018)	Acute dementia ward in hospital, United Kingdom	Health, wellbeing, reducing stress, sense of control and choice, personalised space. Wants needs and preferences	A homely feel, relaxing atmosphere, ability to personalise space, lighting, ventilation, promotes social interaction, safe space (reduces falls), uncluttered and untidy, individual rooms, nature, garden access	Faster healing thus reduced admission time, user satisfaction
18	Clinton‐McHarg et al. (2021)	Treatment centre for cancer, Australia	What person/patients want, needs and their experiences. Wellbeing, mood, perceptions of care	Clean, maps and signs, smell, comfortable furniture, phone access and reception, windows and natural light, uncrowded waiting rooms, clocks, quiet, calm and relaxing colour scheme, private spaces, music. Distractions such as television, reading materials	Physical clutter and person/patient safety, also impacting upon participation. If patient perceived area was cluttered – they would also experience low mood
19	Kelly et al. (2022)	Acute care 100% single room environment, United Kingdom	Privacy, dignity, participation	Single room environment improved access to person/patient, facilitating nursing processes and tasks Room design allowed for more direct care, meaningful time‐ which enhanced decision‐making	Hotel culture, time spent with person/patients, ownership of the space
20	Last et al. (2022)	Stroke rehabilitation, Canada (inpatient and outpatient)	Person/patient participation, personalised care	Noise and disruptions were of concern to person/patients, particularly to rest and sleep Being able to see and interact with other person/patients	Resource availability Healing
21	LaVela et al. (2015)	Veterans Affairs hospitals, United States of America	Quality of care, satisfaction	Upkeep and care of facility, orientation, green spaces, interior space, spatial comfort, lighting, privacy, quiet	Environment can help to calm person/patient, help them cope and thrive
22	Patterson et al. (2017)	Medical surgical ward, USA	Reducing person/patient stress, increase coping	Room layout facilitates views of interest, privacy, technology control, of environment, outlets, bed close to toilet, sliding bathroom door, lighting, storage space. Sense of personal space	Person/patient satisfaction, calming space, helping to cope. Sense of safety. A feeling of being able to heal and sleep
23	Patterson et al. (2019)	Medical surgical ward, USA.	Supporting needs and expectations, person/patient satisfaction, privacy, better sleep, better communication and support	Room layout‐ family zone separate from clinical space, seating for visitors, surface can be personalised, storage, control of television, electric outlets, lighting, overnight accommodation for family	Control of environment Person/patient satisfaction, calming space, helping to cope. Sense of safety. A feeling of being able to heal and sleep
24	Real et al. (2017)	Urban trauma hospital, USA	Communication, increased time and direct person/patient care	Decentralised nurses' stations improved communication with person/patients, visibility and interprofessional communication	New processes and old ways. Nurses perceived decentralised stations as negatively impacting teamwork, communities of practice and increasing their isolation
24	Still et al. (2025)	People living with dementia who are hospitalised to an acute ward (trauma/medical surgical, orthopaedic, family practice, neuroscience/stroke)	Inclusion of partners and family in the care of people in the acute setting	Apace dedicated for care partners to remain in the room during care Environmental resource utilisation Cushioned chairs Waiting rooms, telephone and television	Involving care partners keeps people who live with dementia calm and safe Increased experience
**Mental health**					
26	Lundin (2021)	Psychiatric wards, Sweden	Healing, stress reduction	Aesthetics, bed location, nurses' station, garden space	Tensions between staff need to ensure safety, and a person's right to privacy
27	Olausson et al. (2021)	Forensic psychiatric hospital, Sweden	Safe, calm, ease. In this context‐ supports rehabilitation and recovery	Windows, television and computers, shelves, desk and wardrobe, personal items, light, colour and design, own bathroom. Promotes privacy, sense of self, connection to life	Dual purpose—sense of connection for the person, restorative for offender Calm, harmony
**Outpatient care**					
28	Ajiboye et al. (2014)	Ambulatory outpatient clinic, USA	Relationship‐based care, engagement in care and shared decision‐making	Consultation room layout was changed and both physician and person/patient had equal access to computer screen and results	Paternalism in healthcare, addressing person/patient as active actor in care
29	Almquist et al. (2009)	Outpatient clinics, USA	Relationship‐based care, access to information, shared communication and decision‐making	Consultation room layout was changed to support all people looking at screen. The way people experience care significantly improved	Paternalism in healthcare, control of medical records and information. Passive person/patient

## 4 Findings

**FIGURE 1:**
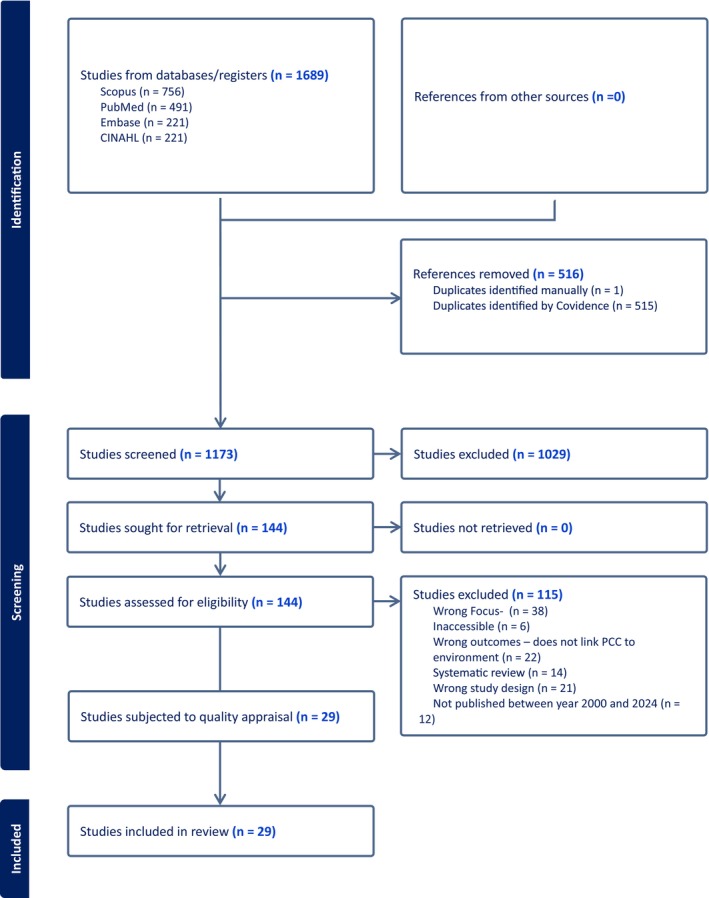
PRISMA search process.

Figure 1 demonstrates the PRISMA process.

A total of 1689 articles were exported for review directly into Covidence (Covidence 2024). Firstly, 516 duplicates were removed, and 1173 articles were included for first‐round review. Subsequently, 144 articles were selected for full text review, with 29 articles being selected for final inclusion.

Of the 29 articles selected for the review, seven were related to aged care contexts, three to paediatrics/neonatal, two to maternity care, ten fell under the broad category of acute care, two were mental health, and the final two related to outpatient settings. Articles included qualitative, quantitative, and mixed methodologies.

The selected literature suggested person‐centred care, as a concept, was related to autonomy and decision‐making, individual needs, preferences and wellbeing, respectful relationships, privacy, dignity, and holistic personhood. In some cases, person‐centred care was described as reducing stress and increasing coping (Lundin 2021; Olausson et al. 2021; Patterson et al. 2017) and improving experience and satisfaction (Annemans et al. 2016; Astin et al. 2020; Kotzer et al. 2011; LaVela et al. 2015; Nielsen and Overgaard 2020; Patterson et al. 2019).

Within the selected literature, person‐centred care in the physical environment was related to aesthetic interventions (green spaces, colour, activities, murals, television, toys, homely feel) and consumer‐based comfort and control (privacy, noise control, wayfinding, controlling lights and temperature, personalization of the space, cooking facilities, security). Two articles discussed person‐centred care environments as facilitating family autonomy (Feeley et al. 2020), decentralised nurses' stations were discussed in one article (Real et al. 2017), and two articles discussed the redesign of the office to facilitate information sharing (Ajiboye et al. 2014; Almquist et al. 2009).

Table 3 provides a summary of articles included and common person‐centred descriptors, the relationship between environments and person‐centred care, and the discursive subtext of the article, whereby the subtext extracts the positioning and underlying premise of the article. Overall contexts and themes are summarised in Table 4. While there is some overlap of subthemes by clinical context, the article presents the findings by clinical context to express the nuanced and changing roles of the physical environment by context.

**TABLE 4:** Summary of findings by context.

Aged Care	The ‘Safety Gaze’
	Reconciling safety to support an environment of personhood
	Nature and stimulation
Paediatrics and neonatal	Supporting parenthood
	Person‐centred care (un)supported through control
	Satisfaction and experience
Maternity	Sterility versus person‐centred care
	Controlling the birth experience
Acute Care	Participation in the environment
	First and foremost a healing environment
	Decentralised nurses station and single rooms
Mental Health	Safety versus person‐centred care
	Calming the environment
Outpatient	Genuine access to information

### 4.1 Aged Care

#### 4.1.1 The Safety Gaze

Those receiving aged care services are often vulnerable and may live with cognitive conditions; thus, safety featured dominantly in the literature. The need for safety is discussed as nurses' visibility (Shannon et al. 2018) and clear space for residents to wander under supervision, without fear of harm (Boumans et al. 2022; Mobley et al. 2017), representing a ‘safety gaze’.

Safety was often described as wayfinding, re‐routing and orientating residents (Catt and Giridharan 2018; De Boer et al. 2018; Mobley et al. 2017), allowing people freedom to move (Boumans et al. 2022) and the environment to sustain independence (Boumans et al. 2022; De Boer et al. 2018). Similarly, literature suggested that lighting, interior design (Mobley et al. 2017) and sensory stimulation are important (Beuls et al. 2024; Chaudhury et al. 2017; De Boer et al. 2018), though this was often linked with reducing aggression, absconding, limiting distress, and improving coping behaviours (Chaudhury et al. 2017; De Boer et al. 2018; Mobley et al. 2017), rather than person‐centred care. While some literature did discuss the environment having an impact on medication use and wellbeing (De Boer et al. 2018), it was often dominated by the overarching need for safety.

#### 4.1.2 Reconciling Safety to Support an Environment of Personhood

Aged care is a unique care setting; the facility becomes the person's home, and considered negotiation between clinical care and homeliness is required. Recognising the need for safety within the context of ‘home’ was raised in the literature, and this was expressed as a reconciliation.

Homeliness and a home‐like space were expressed as important for autonomy (Chaudhury et al. 2017; Van den Berg et al. 2020), and socialisation with other residents and family (Boumans et al. 2022; Chaudhury et al. 2017) instilling a sense of community (De Boer et al. 2018).

A delicate balance between the safety gaze and that of privacy was expressed (De Boer et al. 2018; Shannon et al. 2018). Examples of person‐centred elements included swipe keys to access areas, promoting a sense of privacy and independence (Boumans et al. 2022), and the ability to personalise spaces for the individual (Catt and Giridharan 2018). Likewise, meaningful activities that maximise functioning (such as cooking) were discussed (De Boer et al. 2018; Mobley et al. 2017). Importantly, the role of leadership in establishing and maintaining the marriage between safety, autonomy, and personhood was described, with Catt and Giridharan (2018) describing it as essential to person‐centred care. Sensory elements anchor home‐like elements such as the smell of cooking (De Boer et al. 2018).

#### 4.1.3 Nature and Stimulation

In terms of a person‐centred environment, green spaces and a connection with nature in the space were discussed (De Boer et al. 2018; Van den Berg et al. 2020). Interestingly, nature was described as having a calming effect, providing sensory stimulation, an avenue for social connection (De Boer et al. 2018; Van den Berg et al. 2020) and allowing for healing (Van den Berg et al. 2020).

Freedom to stroll (rather than wander) was deemed important (Mobley et al. 2017), thus, an open space to facilitate movement is warranted. Staff express this as an uncluttered and tidy space, rather than an open, natural one (Catt and Giridharan 2018).

Stimulation also came in the form of farmlands, a novel approach to care which integrates animals and green‐lands (De Boer et al. 2018) and activities and spaces to socialise (Beuls et al. 2024; Chaudhury et al. 2017; Mobley et al. 2017). Notably, Van den Berg et al. (2020) found staff often perceived green spaces to be messy and to add additional duties to their work; however, the literature generally positioned nature and stimulation as a positive element for wellbeing.

### 4.2 Paediatrics and Neonatal

#### 4.2.1 Supporting Parenthood

The desire to support parenthood was described as positioning parents and caregivers as custodians of care and decision‐makers (Feeley et al. 2020), often through single rooms. Single rooms were discussed as a way of preparing families for discharge (Feeley et al. 2020). The unique needs of patients and caregivers were also noted and supported through consultation rooms and play spaces (Pattabi et al. 2024).

#### 4.2.2 Person‐Centred Care (Un)Supported Through Control

In this context, person‐centred care was associated with an environment where parents could practice with their child, make decisions, and feel connected as a family (Feeley et al. 2020), mostly through having the privacy of a single room which kept the family unit together. Creature comforts, such as a washing machine and kitchen, were designed to offer convenience, freedom and control in the environment. A safe carpark was purported to have an impact on feelings of control (Kotzer et al. 2011).

Within the immediate environment, control of the temperature of a room, lighting and noise was discussed as important, and reduced stress in parents and families during difficult times (Kotzer et al. 2011). Design elements such as toys, colourful and attractive play areas, signage and televisions were listed as important (Pattabi et al. 2024).

Studies detailed staff perceptions of ward redesign, including single rooms and decentralised nursing stations. Interestingly, staff did not necessarily support such design choices, feeling they disrupted workflow, increased distance walked, and removed senses of support and comradery often facilitated through a centralised station (Feeley et al. 2020; Kotzer et al. 2011).

#### 4.2.3 Satisfaction and Experience

A clear theme in the literature centered around improving satisfaction and experience through design choices. Articles discussed wall colour and décor, wayfinding signage (Pattabi et al. 2024), art and lounges, and making the environment less scary for children (Kotzer et al. 2011). This was framed around wellbeing and mood; however, the literature meandered between the ideas of person‐centeredness and patient satisfaction.

### 4.3 Maternity

#### 4.3.1 Sterility Versus Person‐Centred Care

The birth experience is often considered in relation to the dichotomy of medical intervention and natural process. As such, the sterile and medicalized nature of the birthing suite was discussed in relation to person‐centeredness. Sheehy et al. (2011) described this as sterility versus a homely experience.

Environmental factors that broke up the clinical environment included welcoming spaces, audio and visual stimulation, sofas, bathtubs and colours and textures on walls (Nielsen and Overgaard 2020; Sheehy et al. 2011), along with privacy and the use of birth balls, having an en‐suite bathroom, and feeling safe within the space (Nielsen and Overgaard 2020; Sheehy et al. 2011).

#### 4.3.2 Controlling the Birth Experience

For women and families, having control over and within the environment was important. Single rooms were viewed as important for privacy and keeping families together. Specifically, having a space for partners or family to remain with the birthing person was important. Control over lighting and room noise was also discussed (Nielsen and Overgaard 2020; Sheehy et al. 2011). In line with the paediatrics and neonatal context, the relationship between the environment and person‐centred care built satisfaction, a positive experience, and offered distraction from clinical processes (Nielsen and Overgaard 2020; Sheehy et al. 2011).

### 4.4 Acute Care

For the purposes of this article, acute care was considered an inpatient setting where care was provided along a time‐limited trajectory.

#### 4.4.1 Participation in the Environment

Participation in the environment was seen as important to person‐centred care. Articles referred to patients having control over the door to their room, being able to see who is coming, controlling lighting, and having easy access to one's belongings (Annemans et al. 2016; Clinton‐McHarg et al. 2021; Douglas and Douglas 2004). Controlling noise, temperature, and lighting was also important (Astin et al. 2020; Douglas and Douglas 2004; Last et al. 2022; Patterson et al. 2017, 2019). Having a single room and privacy was seen as necessary for person‐centred care (Kelly et al. 2022; LaVela et al. 2015; Patterson et al. 2019). Such control was described as reassuring for patients, providing comfort, autonomy and improved experience of care (Buchwald et al. 2024; Clinton‐McHarg et al. 2021; Douglas and Douglas 2004; LaVela et al. 2015; Real et al. 2017). Similar design elements to other contexts, such as greenspaces (LaVela et al. 2015) and entertainment and distraction (Clinton‐McHarg et al. 2021), were also evident in the literature. Dedicated family spaces were also viewed as important, particularly where people experienced cognitive or functional decline (Buchwald et al. 2024; Still et al. 2025).

Visitor engagement in this setting was also described as important to person‐centred care. Visitor seating and comfort, and the ability for participation via an open layout were discussed (Douglas and Douglas 2004; Patterson et al. 2019).

#### 4.4.2 First and Foremost a Healing Environment

Improving physical spaces, for example, single rooms, is discussed in the literature as designed to improve meaningful staff/patient time (Kelly et al. 2022) with the intent of healing (Buchwald et al. 2024; Douglas and Douglas 2004). The healing environment is conditional to safe practice; thus, safety was again prominent (Annemans et al. 2016; Douglas and Douglas 2004). Decluttered spaces were described as important for safety (Still et al. 2025; Clinton‐McHarg et al. 2021).

Several design elements were discussed including the use of handrails, doors which are easily opened, easy‐to‐use curtain rails (Patterson et al. 2019), temperature and noise control (Astin et al. 2020; Douglas and Douglas 2004; Last et al. 2022), clocks and calm/relaxing spaces (Clinton‐McHarg et al. 2021). Interestingly, some articles discussed the physical environment having a relationship with staff processes and workflows (Douglas and Douglas 2004), for example, separation between the family/visitor zone and the clinical zone, allowing staff to prioritise work (Patterson et al. 2019).

However, the literature raised a tension about the perceptions of staff in relation to person‐centred design elements. Kelly et al. (2022) raised the idea of ‘hotel culture’ where person‐centred care positioned service over care, leading to negative staff feelings toward the concept. Indeed, the literature alluded to perceived declines in clinical care (Douglas and Douglas 2004) and ownership of the space (Kelly et al. 2022). Staff seemed to value cleanliness and lack of clutter, safety and visibility, over patient control of the environment (Annemans et al. 2016; Clinton‐McHarg et al. 2021; LaVela et al. 2015; Real et al. 2017), thus reinforcing the hospital as a medical institution rather than one concerned with experience or satisfaction.

#### 4.4.3 Decentralised Nurses Station and Single Rooms

One common person‐centred design feature was decentralised nurses' stations and single rooms, with many tensions within the literature raised around this. Decentralised nurses' stations could improve communication and time between nurses and those they care for; however, it was also reported to negatively impact teamwork and lead to isolation from peers (Real et al. 2017). Real et al. (2017) suggested that not having a central area led to nurses spending increased time looking for medical officers and people/patients spending more time looking for nurses, posing safety issues, increasing walking time, and reducing staff education time. There was some suggestion that single rooms meant nurses were more process or task‐orientated rather than using a person‐centred approach (Kelly et al. 2022). Other healthcare staff did not necessarily agree, believing decentralised spaces to be positive, keeping staff close to patients (Real et al. 2017). Single rooms were generally viewed favourably by those receiving care (Kelly et al. 2022).

### 4.5 Mental Health

#### 4.5.1 Safety Versus Person‐Centred Care

The literature discussed the need for safety within the mental health environment, as a form of care (Lundin 2021; Olausson et al. 2021). Safety seemed to be the dominant concern for staff and was viewed at odds with person‐centred care (Lundin 2021). Olausson et al. (2021) suggested a safe environment made patients feel safe; however, the costs of this included lack of privacy, no access to computers, and loss of freedom, identity and an inability to make decisions. Lundin (2021) described some attempts to make the space aesthetically pleasing, homely, with a good bed.

#### 4.5.2 Calming the Environment

Attempts to make the space pleasing also seemed to serve the dual purpose of calming the patient. For example, windows, televisions, shelves and desks, good lighting, colours and designs (Olausson et al. 2021), greenspaces and the position of the bed in the room (Lundin 2021) were described as creating harmony and reducing tensions. In the mental health context, this was viewed as important.

### 4.6 Outpatient

#### 4.6.1 Genuine Access to Information

The outpatient literature on person‐centred care and the physical environment was particularly interesting. Both articles (Ajiboye et al. 2014; Almquist et al. 2009) discussed the physical layout of outpatient rooms. Changes were made to room design to reduce the physical barrier of the medical officer desk. People/patients and staff sat on the same side of the desk with equal access to the computer screen. This simple intervention was designed to facilitate genuine information sharing and transparency (Ajiboye et al. 2014; Almquist et al. 2009). Furthermore, the intervention addressed the underlying understanding that healthcare is dominantly paternalistic, where information is tightly controlled and the patient is positioned as a passive actor. The act of sitting beside the staff and reviewing information together improved how people experienced the care they received (Ajiboye et al. 2014; Almquist et al. 2009).

## 5 Discussion

This review aimed to explore literature in relation to the physical healthcare environment and person‐centred care to understand how it can be better supported at the system level. It found consistencies across the literature despite the context of care, including the perceptions of safety and risk, and the idea of ‘hotel culture’.

Firstly, the literature shows attention has been paid to multiple design elements across different contexts globally. This is positive for hospital and health services; gone are the days of clinical white walls and sterility of hospitals. However, a focus has been placed on aesthetics and satisfaction rather than genuine person‐centred care.

Hospital design moves with the ebbs and flows of societal and cultural norms of health and of care. Bromley (2012) describes this as an interpretive act, where such trends are influenced by the rise in healthcare consumerism and the embedding of business principles into care. Bromley (2012) carefully articulates that hospital design reflects the values and aspirations of hospital managers and leaders, forming a social identity.

Prominent in the literature was the need to manage safety and risk, and that this often battled person‐centred care. In areas such as aged care, safety was discussed in terms of risk and liability. Staff often experience tensions between facilitating certain freedoms, such as open doors and encouraging independent walks, when there are continual safety risks with lacking visibility. Staff are often concerned with the consequence of not maintaining a patient's safety and about potential blame or liability (Mulvogue 2024). However, there are benefits to enabling the person to freely move between rooms and continue activities that sustain cognitive functioning and reduce mental health challenges (Woods et al. 2023). Environments with sensory stimulation feed memories, encouraging an atmosphere of ‘home’. Conversely, low staffing levels, tight schedules, legal responsibilities, and task‐oriented care are a sad reality for many facilities (Ludlow et al. 2020), often resulting in person‐centred care being overlooked and replaced by the control of the ‘safety gaze’ to reduce risk and injury. Initiatives centre around keeping patients calm and busy to minimise harm in what is largely viewed to be an unsafe environment (Shannon et al. 2018), highlighting how staff perceptions can influence person‐centred care.

The same can be said for many of the contexts this review explored. Safety, injury prevention, and systemized care seem to permeate all aspects of the environment, including philosophies of design, purpose and physical space. While design principles may be person‐centred at heart, the characteristics, challenges and policies of environmental design are often not included in hospital and health service strategic plans, and thus are not moved forward (Elf et al. 2015).

Making change is difficult. Van den Berg et al. (2020) found changes to the environment were interpreted as ‘messy’ by staff, adding burden to their work. To some degree, it is natural that staff would focus on implications to practice in any healthcare change (including design); however, the notion of person‐centred care is not new, and this highlights a tension between safety and centeredness. This demonstrates two things: firstly, notions of quality and safety in healthcare are well embedded and permeate the decisions and practices of staff and the organisation. Secondly, focus on quality and safety measures often translates to task orientation (e.g., completing paperwork by prescribed times) (ACSCQH 2024a, 2024b). Task orientation is the antithesis of person‐centred care, satisfying the needs of staff and organisations rather than individuals (Kwame and Petrucka 2021). The literature in this review supports this, finding design elements and changes which disrupted traditional workflows and team cohesion were viewed unfavourably. This is, of course, driven by culture.

The culture of medical care, the hospital, and the associated environment are sociologically driven, meaning a specific script exists of what a hospital should be. Stemming from historical roots, the hospital is a physical place and an institutionthat holds ideals and values. Martin et al. (2015) argued that the rise of new public management and the commodification of health opened the space for architectural design features of the hotel, an obfuscation of the boundaries of the hospital into the business of medicine. Only recently has architecture served as a symbol of this (Sloane and Sloane 2003). This ‘anti‐institutional’ aesthetic ensures that form and function are interwoven; a sign of the times and an indication of the business of healthcare (Martin et al. 2015). While the intent may be to ‘humanise’ an historically sterile experience, we cannot undervalue the social, political, and economic trends that support such redesign (Bates 2017).

Healthcare staff are increasingly subjected to low staffing levels, rationed care, resources, and financial limitations (Scott et al. 2019). A key motivator for nurses is maintaining a manageable workload and the operational functioning of a facility, thus maintaining the status quo. Integration of design features intentionally aimed at enhancing comfort and reducing the sterile feel of the healthcare setting can leave staff feeling unsettled, as the focus of the space is to serve patients, not clinicians (Kotzer et al. 2011). Staff at times buck this transition, feeling that people/patients use services as ‘hotels’, rather than hospitals. These findings highlight that many staff feel more at ease in a clinical space constructed to facilitate their work, allowing a greater sense of control over the environment (see Feeley et al. 2020). Indeed, healthcare staff seem to lament the transition to consumerised ‘hotel culture’. Changes in design are perceived by staff as disruptive to teams, increasing workloads, complicating daily work routines, and cluttering spaces (Catt and Giridharan 2018; Feeley et al. 2020; Kotzer et al. 2011; Van den Berg et al. 2020). These perceptions may not be without reason, with Bromley (2012) describing the marketing of health and wellbeing, which acts to instill the commodity of health in the urban landscape. The hospital itself is designed with onstage/offstage principles, where care becomes hidden under the aesthetic, providing a ‘Disneyland’ experience (Bromley 2012) for consumers. It is understandable that tensions between the aesthetic and the purpose of the hospital are voiced by staff.

Indeed, person‐centred care, practice within the care environment, represents a complexity that requires cohesion and support across all levels. This is articulated in the Person‐Centred Nursing Framework (McCormack and McCance 2025) and other frameworks, which are careful to articulate the metaparadigms of nursing, the structures, cultures, and people within it. Processes are driven by, among many things, the environment in which care exists, necessitating cohesion of person‐centeredness across the hierarchies and structures that exist in healthcare. The literature blurs the lines between person‐centred care and patient satisfaction/experience, and while certainly some congruence may exist, person‐centred care as a system responsibility is about genuine engagement and inclusion in care (Byrne 2024).

This review aimed to explore person‐centred care and the physical environment, seeking ways for healthcare systems to improve. An avenue worthy of further exploration is a focus on design supporting *genuine* partnership. Healthcare workers are in the unenviable position of doing more with less, caring for increasingly complex patients in ill‐designed systems. The need to ‘comply’ with system requirements under the safety and quality movement adds an additional layer, with the potential to translate into task orientation and the desire to strongly control the environment. Tasking healthcare workers with a need to practice person‐centred care and changing the facility aesthetic does not constitute a person‐centred environment.

Of 29 included articles, only four are congruent with genuine facilitation of engagement and inclusion; the others, while important, related to satisfaction. Person‐centred care design principles in most articles were aesthetic and revolved around comfort. Articles that discussed autonomy, shared decision‐making, and collaboration were few; single rooms were discussed as facilitating family autonomy and decision‐making (Feeley et al. 2020), decentralised nurses' stations kept healthcare staff close to patients in the ICU (Real et al. 2017), and the redesign of outpatient offices to facilitate information sharing was discussed in two articles (Ajiboye et al. 2014; Almquist et al. 2009). These are examples of genuine person‐centred care, and the limited studies on interventions at this level demonstrate that healthcare systems have some way to go in supporting person‐centred care. This opens a space to ask, ‘what else is missing?’ Some elements of person‐centred care were absent, including coordination of care and efforts to reduce fragmentation. These are significant healthcare issues, which require structural, cultural, economic and environmental change.

This review found that efforts to support person‐centred care within the physical healthcare environment have commenced (at a surface level), albeit reflecting the cultural and social shift of consumerism in healthcare. While these interventions are no doubt effective in creating positive experiences for those receiving care (and their families), they are somewhat superficial in achieving genuine, system‐led person‐centred care. Healthcare must move beyond satisfaction and seek more meaningful ways to support engagement, inclusion, autonomy, and decision‐making. Research into co‐designed environment changes is sorely needed, particularly that which facilitates information sharing and decision‐making for those receiving care. The work of Feeley et al. (2020), Real et al. (2017), Ajiboye et al. (2014) and Almquist et al. (2009) provides significant insights into how genuine PCC might be facilitated through the environment. Additionally, the time is right for local policymakers, accreditors, and healthcare leaders to advance person‐centred care, ensuring it is true to its purpose and philosophy of care, seating people/patients as drivers.

## 6 Limitations

This review was conducted with published literature only, and the way this is interpreted and enacted in health services was not included. This decision was made due to the sample size of 29, allowing for a deep analysis of the published literature alone. However, grey literature may have provided more local insights into the physical environment; hence, specific nuance may have been missed.

Some contexts had limited articles included; thus, themes may not be generalizable.

## 7 Conclusion

This review aimed to ascertain how the physical care environment impacts upon person‐centred care, with a specific aim of looking away from practice. The review found unique themes dependent on the context of care; however, more generalizable categories were evident outside of context, namely the perceptions of safety and risk, and the notion of ‘hotel culture’. While described as person‐centred, many design elements were more about satisfaction than engagement and decision‐making. Person‐centred design in hospitals makes for a more aesthetically pleasing environment and positive experience; however, these elements are superficial in nature regarding genuine person‐centred care. Healthcare policymakers, accreditors, and leaders must move beyond satisfaction and consider environmental changes which genuinely engage people in their care and promote shared decision‐making, including information sharing, engagement, coordination, and collaboration.

## Funding

The authors have nothing to report.

## Ethics Statement

As a systematic literature review, no ethics were required for this research.

## Conflicts of Interest

The authors declare no conflicts of interest.

## Supporting information


**Appendix S1:** nop270395‐sup‐0001‐AppendixS1.docx.

## Data Availability

The data that supports the findings of this study is available in the Appendix S1 of this article.

## Appendix A

Example Search String

((‘Person cent* care’[Title/Abstract] OR ‘patient cent* care’[Title/Abstract] OR ‘family cent* care’[Title/Abstract] OR ‘Client Cent* care’[Title/Abstract] OR ‘resident cent* care’[Title/Abstract] OR ‘woman cent* care’[Title/Abstract]) AND (‘Physical environment*’[Title/Abstract] OR ‘health* structure’[Title/Abstract] OR infrastructure[Title/Abstract] OR surroundings[Title/Abstract] OR ‘practice environment’[Title/Abstract] OR ‘facility design and construction’[Title/Abstract] OR ‘hospital design’[Title/Abstract])) AND (Hospital[Title/Abstract] OR ‘health clinic’[Title/Abstract] OR ‘health sector’[Title/Abstract] OR ‘nursing home’[Title/Abstract] OR ‘health service’[Title/Abstract] OR ward[Title/Abstract] OR ‘inpatient service’[Title/Abstract]).
